# Light Color and the Commercial Broiler: Effect on Ocular Health and Visual Acuity

**DOI:** 10.3389/fphys.2022.855266

**Published:** 2022-03-10

**Authors:** Bruna Remonato Franco, Marina L. Leis, Melody Wong, Tory Shynkaruk, Trever Crowe, Bryan Fancher, Nick French, Scot Gillingham, Karen Schwean-Lardner

**Affiliations:** ^1^Department of Animal and Poultry Science, University of Saskatchewan, Saskatoon, SK, Canada; ^2^Department of Small Animal Clinical Sciences, Western College of Veterinary Medicine, University of Saskatchewan, Saskatoon, SK, Canada; ^3^Department of Ophthalmology, Saskatoon City Hospital, University of Saskatchewan, Saskatoon, SK, Canada; ^4^Department of Mechanical Engineering, University of Saskatchewan, Saskatoon, SK, Canada; ^5^Aviagen™ Inc., Cummings Research Park, Huntsville, AL, United States

**Keywords:** broiler, wavelength, light color, spatial vision, visual function

## Abstract

Light is a critical management factor for broiler production, and the wavelength spectrum, one of its components, can affect bird physiology, behavior and production. Among all the senses, sight is important to birds, and their visual system possess several adaptations that allow them to perceive light differently from humans. Therefore, it is critical to consider whether the exposure to monochromatic light colors influences broiler visual ability, which could affect behavioral expression. The present study examined the effects of various light colors on the visual systems of broiler chickens. Ross 708 males were raised from 0 to 35 days under three wavelength programs [blue (dominant wavelengths near 455 nm), green (dominant wavelengths near 510 nm) or white]. Broilers were given a complete ophthalmic examination, including chromatic pupillary light reflex testing, rebound tonometry, anterior segment biomicroscopy and indirect ophthalmoscopy (*n* = 36, day 21). To assess ocular anatomy, broilers were euthanized, eyes were weighed, and dimensions were taken (*n* = 108, day 16 and day 24). An autorefractor was used to assess the refractive index and the corneal curvature (*n* = 18, day 26). To evaluate spatial vision, broilers underwent a grating acuity test at one of three distances–50, 75, or 100 cm (*n* = 24, day 29). Data were analyzed as a one-way ANOVA using the MIXED procedure or Proc Par1way for non-normally distributed data. Significant differences were observed for refractive index and spatial vision. Birds raised under blue light were slightly more hyperopic, or far-sighted, than birds raised under white light (*P* = 0.01). As for spatial vision, birds raised under blue light took less time to approach the stimulus at distances of 50 cm (*P* = 0.03) and 75 cm (*P* = 0.0006) and had a higher success rate (choosing the right feeder, *P* = 0.03) at 100 cm than birds raised under white light. Improvements in spatial vision for birds exposed to blue light can partially explain the behavioral differences resulting from rearing broilers under different wavelengths.

## Introduction

Light is a crucial management factor for broiler production, and its components, such as photoperiod and light intensity, affect broiler growth, diurnal rhythms, behavior and welfare (Deep et al., 2010, 2012; Schwean-Lardner et al., 2012a,b, 2014). Light spectrum, another aspect of light, appears to affect poultry growth; however, the results published to date are inconsistent (Prayitno et al., 1997a; Rozenboim et al., 1999; Mohamed et al., 2017). It is well documented that behavioral responses are altered when birds are reared under specific light wavelengths (Prayitno et al., 1997a,b; Lewis and Morris, 2000; Sultana et al., 2013; Mohamed et al., 2017). Previous studies have indicated that raising broilers under long wavelengths, such as red light (630–780 nm), increased bird activity, with increased walking and wing and leg stretching (Prayitno et al., 1997b). Broilers were also more active when raised under yellow light (565–600 nm), which is also considered a long wavelength (Sultana et al., 2013). In contrast, birds raised under shorter wavelengths, such as blue light, spent more time sitting or resting (Prayitno et al., 1997a; Sultana et al., 2013). In addition to the impacts on behavioral output, light wavelength also influences fear and stress levels. Broilers raised under blue light had lower fear levels, assessed through tonic immobility, than birds raised under white light (Mohamed et al., 2014, 2017). Likewise, the heterophil: lymphocyte ratio, an indicator of chronic stress, was lower in broilers reared under blue light compared to green or white light, indicating a reduction in stress (Remonato Franco et al., under review a^1^).

These impacts on bird behavior may suggest that lighting programs with varying wavelengths may be a usable tool to improve welfare and production. However, it is important to understand the origin of the behavioral changes and whether they are related to visual ability. A bird’s large eyes in relation to their body weight and brain size suggest that vision is a critical sense for poultry (Garamszegi et al., 2002; Prescott et al., 2003). Birds use visual cues for several activities, such as awareness of other birds’ intentions, status recognition, determining what is safe to eat and drink, and navigation (Collins et al., 2011). When comparing the behavior of blind and sighted chickens, Collins et al. (2011) demonstrated that blind birds exhibited difficulties in expressing key behaviors, displaying increased frustration, which is associated with reduced animal welfare.

Birds have developed several adaptations to their visual system compared to mammals, despite sharing similar gross features (Bennett and Cuthill, 1994). Birds are tetrachromatic, which means they possess four types of cone photoreceptor cells compared to trichromatic animals, such as humans. The lens and aqueous humor of birds are optically clear in the ultraviolet (UV) range, giving birds a color vision expanded to UV light (Lewis and Morris, 2000; Marshall and Arikawa, 2014). They also possess a double-cone photoreceptor, of which the function is related to the perception of movement (Kram et al., 2010). The oil droplets in the cones filter light before it reaches the photopigments, providing birds with increased accuracy in color discrimination (Prescott and Wathes, 1999; Kelber, 2019). Birds also possess a different spectral sensitivity and therefore perceive color and intensity differently than humans (Prescott and Wathes, 1999; Prescott et al., 2003). This is particularly true in the blue color range, and birds see this light color much brighter than do humans (Lewis and Morris, 2000). This implies that measuring intensity in units of lux, the traditional unit used to assess illuminance under white light, may not be the correct methodology when assessing light intensity of colored lighting in poultry settings, as it is based on human spectral sensitivity rather than that of a bird. A more accurate assessment unit has been developed, known as clux (corrected lux or chicken lux), that considers birds’ spectral sensitivity and how they perceive their environment (Nuboer et al., 1992; Prescott and Wathes, 1999; Lewis and Morris, 2000; Prescott et al., 2003; Kristensen et al., 2006).

Previous research has highlighted the impacts of other light components, such as long daylengths, in broiler (Schwean-Lardner et al., 2013) and turkey production (Vermette et al., 2016). These animals display increased eye weights and dimensions as daylength increases, likely through disruption of the diurnal rhythms, which also happens when light intensity is very low during broiler production (Deep et al., 2013).

Besides affecting eye shape and size, visual function may be impaired by different light components, influencing refraction, ocular health, and visual acuity (Flaxman et al., 2017). The chicken eye has been used as a model for studying human ocular diseases and conditions (Wisely et al., 2017). Results demonstrate an immoscopy was performed to assess thpact of varying wavelengths on emmetropization, with eyes exhibiting increased axial length and vitreous chamber depth when chicks were exposed to red or white light (Lin et al., 2020), and on refractive error, with long wavelengths, such as red light, leading to progressive myopia (Foulds et al., 2013). To our knowledge, no study has been conducted to understand the structural ocular changes or effects in visual function and spatial vision caused by raising broilers under different wavelengths in a simulated commercial setting.

To encompass this, in the present study, broilers reared under one of three wavelengths underwent a series of tests to assess their visual ability. These data were of a larger experiment, testing the response to wavelength treatments in production, health and behavior parameters of broilers (3 manuscripts: Table 1. see text foot note 1^2,3^). The objective of this study was to assess ocular health and vision in broilers raised under different wavelength treatments.

**TABLE 1:** Summary of experiments/trials and measurements obtained from each.

Current manuscript			Other measures taken within specific experiment
Experiment 1	Trial 1	*Ocular health* (chromatic PLR, anterior segment biomicroscopy, indirect ophthalmoscopy, IOP) *Eye measurements* (eye weight, corneal diameter, mediolateral diameter, dorsoventral diameter and anteroposterior size)	Behavioral expression, fear and stress levels (see text foot note 1) Production variables (see text foot note 2) Health parameters (see text foot note 3)
	Trial 2	*Refraction Index* *Eye measurements* (eye weight, corneal diameter, mediolateral diameter, dorsoventral diameter and anteroposterior size)	
Experiment 2		*Spatial vision*	

## Materials and Methods

This experiment was approved by the Animal Care Committee of the University of Saskatchewan and was conducted following the guidelines of the Canadian Council on Animal Care (1999) as specified in the Guide to the Care and Use of Experimental Animals.

The assessments of ocular health and vision were conducted as a part of a larger experiment that studied the impact of wavelength treatments on production, physiology, behavior and welfare of broilers. Tests were conducted within 2 experiments, one of which involved 2 blocked trials.

### Housing

For both experiments, each lasting 35 days, broilers were raised at the Poultry Research and Teaching Unit located at the University of Saskatchewan. The facility contains nine individual environmentally controlled rooms. Each room was subdivided into 12 individual pens (2.0 m × 2.3 m). In Experiment 1 (2 repeated trials), Ross YPMx708 and Ross EPMx708 males and females were housed sex-separately within nine rooms (12 pens per room), with 62 males, or 70 females per pen, resulting in a final estimated density of 31 kg/m^2^ (total of 7128 broilers housed in each of two trials). For Experiment 2, mixed-sex Ross 308 chicks were housed in two rooms, with each pen containing 42 broilers, for a total of 710 broilers.

Water, available *ad libitum*, was provided using pendulum nipple drinkers, with six nipples available per pen. Commercially prepared feed (starter diet–0.5 kg per bird, grower diet–2.0 kg per bird and the remainder as finisher diet) was provided *ad libitum* using aluminum tube feeders. Chicks were housed on the day of hatch in pens containing approximately 7.5–10 cm of wheat straw bedding. Room temperatures were set to 32.1°C at the time of placement and were reduced gradually until 21°C was reached by 25 days of age and maintained until the end of the trial. Temperature was monitored twice each day in each room *via* behavioral observations and computer output.

### Lighting

**FIGURE 1:**
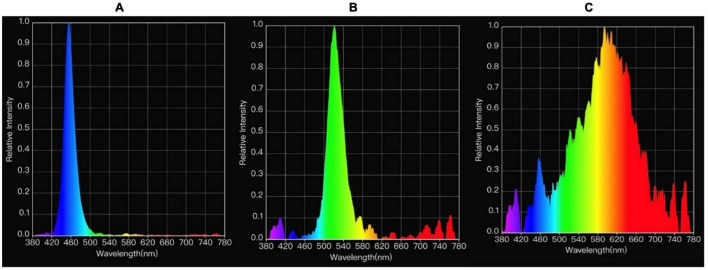
Measurements of light spectrum, respectively, from blue **(A)**, green **(B)** and white **(C)** light treatments.

Light was provided *via* light emitting diode (LED) light bulbs (11W Alice Non-Directional LED Lamps, Greengage Agritech Limited, Roslin Innovation Centre, University of Edinburgh, Easter Bush Campus, Midlothian, EH25 9RG, United Kingdom) that, for Experiment 1, emitted one of three lighting treatments: blue (dominant wavelengths near 455 nm), green (dominant wavelengths near 510 nm), and white (combination of wavelengths). Experiment 2 treatments were refined (chosen based on behavioral differences noted between broilers reared under blue light compared to white and green: see text foot note 1), and included only the blue and white lighting treatments. These were chosen based on behavioral measures taken in Experiment 1 (see text foot note 1). Measurements of light spectra were taken for each light treatment to verify the spectral distribution (Asensetek Incorporation, New Taipei City, Taiwan, Figure 1).

On day 0, birds received 23 h of light, which was decreased (1 h per day) until day 5, when 18 h of light was provided. Dawn and dusk periods of 15 min were provided daily, prior to lights turning on or off. Light intensity was measured in clux. For trial 1 of Experiment 1, light intensity was 9.6 ± 0.4 clux. In the second blocked trial of Experiment 1, and for Experiment 2, the first week was set at 14.3 ± 0.1 clux and the remaining weeks at 9.6 ± 0.4 clux (Galilux Light Meter, Hato Agricultural Lighting, Sittard, Netherlands).

### Data Collection

#### Ocular Health

A summary of the data collected during the different experiments and trials is presented at Table 1. In the first trial of Experiment 1, twelve YPM-708 males per lighting treatment (from 2 pens per room and 2 birds from each pen) were randomly selected at 21 days (*n* = 36) to undergo complete ophthalmic examinations performed by a board-certified veterinary ophthalmologist. The examinations included chromatic pupillary light reflex (PLR) testing (Melan-100 unit, BioMed Vision Technologies, Ames, IA, United States), anterior segment biomicroscopy performed with a portable slit lamp (SL-17, Kowa, Tokyo Japan), and indirect ophthalmoscopy [IOP, (Heine Omega 200, Heine Instruments Canada, Kitchener, Ontario)]. Rebound tonometry (Tonovet, Tiolat, Helsinki, Finland) was used to estimate intraocular pressure (Wahl et al., 2016; Leis et al., 2017).

#### Eye Measurements

In Experiment 1 (trials 1 and 2), 36 birds per light treatment (YPM-708 males only, 2 pens per room, 1 bird per pen, *n* = 108) were euthanized by decapitation, and both eyes were immediately extracted. Adhering tissues were removed, and eye weights and dimensions were taken for both eyes using a digital scale and digital caliper. Dimensions included corneal diameter, mediolateral diameter, dorsoventral diameter and anteroposterior size (Schwean-Lardner et al., 2013; Vermette et al., 2016).

#### Refraction Index

In the second trial of Experiment 1, a handheld autorefractor (Nikon Retinomax K-plus 2) was used to assess the corneal curvature and the refractive index in six birds per lighting treatment at 26 days (YPM-708 males only, from 2 pens per room and 1 bird from each pen, *n* = 18). Tests were conducted and results were assessed by an ophthalmologist from Saskatoon City Hospital, University of Saskatchewan (Saskatoon, SK, Canada).

#### Spatial Vision Test

**FIGURE 2:**
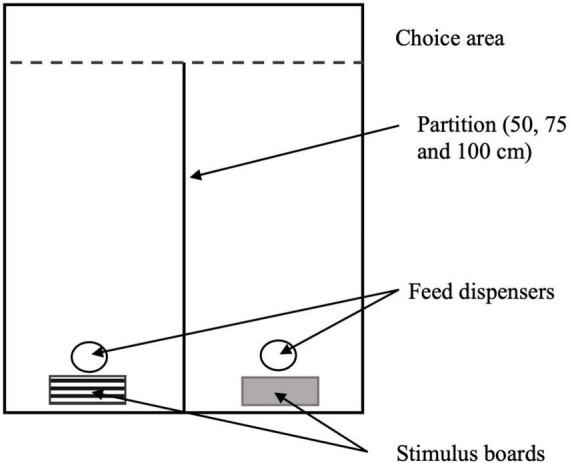
Schematic representation of the test pens, adapted from Kristensen (2004).

Based on behavioral differences noted for birds under blue compared to white and green light in Experiment 1 (see text foot note 1), a second experiment was added to determine if visual acuity could explain these behavioral changes. In Experiment 2, 12 birds per lighting treatment (blue or white, from one pen per room, males only, *n* = 24) underwent testing to evaluate spatial vision at 29 days, using a spatial conditional discrimination procedure (DeMello et al., 1992). For a period of 4 days before the test day, a test space was set up in the middle of a pen, and selected birds were placed individually inside the pen. Birds remained in the test space for 5 min, for acclimation with the features related to rewarding and non-rewarding feeders (Kristensen, 2004). The test pen included two feed stations: one contained previously weighed feed (rewarding) and the other without feed (non-rewarding), Figure 2. The feed stations were located either in front of gray or black and white square-wave grating boards [vertical stripes–2.5 mm wide, 5 mm per cycle (Hyvärinen, 2018)].

On the fifth day of spatial vision testing, the chosen birds were individually tested in the test pens. Each test pen contained both feed dispensers (rewarding and non-rewarding–the same as presented in the training period), placed in front of the gray or grating stimulus boards at the end of the pen (DeMello et al., 1992). The feed dispensers were separated by a partition, which varied in length to test birds at different distances from the stimulus boards. Feed was withdrawn from birds 1 h prior to testing. Birds were individually placed in the “choice area” at one of three different distances at a time (50, 75, and 100 cm from the feeders), where they chose between the two stimuli. The average time taken to approach a feed dispenser and whether animals chose the dispenser that contained feed (success rate) was recorded by an observer, who stayed outside of the vision area of the birds being tested (Kristensen, 2004).

### Statistical Analyses

Data were statistically analyzed using SAS (SAS 9.4, Cary, NC, United States). Bird was used as the experimental unit for all tests. All data were tested for normality before analyses using the UNIVARIATE procedure. Data were analyzed as a one-way ANOVA using the MIXED procedure or PAR1WAY procedure for data not normally distributed. Tukey’s range test was used to separate means when significant differences were found. Differences were considered significant when *P* ≤ 0.05.

## Results

### Ocular Health

Chromatic PLR (cPLR) testing, which indicates retina and optic nerve functions after light stimulus, did not reveal any significant differences between birds reared on various light treatments. Likewise, both anterior segment biomicroscopy and indirect ophthalmoscopy did not reveal abnormalities in any birds, indicating no abnormalities in the fundus of the eye. Intraocular pressure, which, if high, can lead to glaucoma, was within normal limits in all birds and did not differ with the use of blue, green or white light during the production period of broilers (*P* = 0.74, Table 2).

**TABLE 2:** The effect of light color treatments on intraocular pressure (mmHg) of YPMx708 male broilers at 21 days of age (*n* = 36, Experiment 1, trial 1).

	Light treatment				
	Blue^1^	Green^2^	White^3^	*P*-value	SEM^4^
Intraocular Pressure (mmHg)	8.58	8.83	8.88	0.74	0.122

*^1^Blue light–dominant wavelengths 435–500 nm, peak at 455 nm.*

*^2^Green light–dominant wavelengths 500–565 nm, peak at 510 nm.*

*^3^White light–range of wavelengths from 380 to 780 nm.*

*^4^SEM = Standard error of the mean.*

### Eye Measurements

Raising broilers under either blue, green or white lights had no effect on eye weight (*P* = 0.13) or dimensions (shape) of the eye, which, if altered, could impact image forming, including the corneal diameter (*P* = 0.95), mediolateral diameter (*P* = 0.14), dorsoventral diameter (*P* = 0.22) or anteroposterior size (*P* = 0.78, Table 3).

**TABLE 3:** Effect of different light color treatments on left and right eye weight and dimensions of YPMx708 male broilers at 17 days of age (*n* = 108, Experiment 1).

	Light treatment				
	Blue^1^	Green^2^	White^3^	*P*-value	SEM^4^
Eye wt. (g)	1.12	1.14	1.16	0.13	0.001
Eye wt./body wt. (%)	0.03	0.03	0.03	0.51	0.236
Corneal diameter (mm)	6.40	6.39	6.42	0.95	0.029
DV diameter^5^ (mm)	13.94	14.06	14.19	0.22	0.073
ML diameter^6^ (mm)	13.92	13.94	14.16	0.14	0.060
AP depth^7^ (mm)	11.57	11.66	11.52	0.78	0.067

*^1^Blue light–dominant wavelengths 435–500 nm, peak at 455 nm.*

*^2^Green light–dominant wavelengths 500–565 nm, peak at 510 nm.*

*^3^White light–range of wavelengths from 380 to 780 nm.*

*^4^SEM = Standard error of the mean.*

*^5^Dorsoventral (DV) diameter.*

*^6^Mediolateral (ML) diameter.*

*^7^Anteriorposterior (AP) depth.*

### Refractive Index

Testing refraction, which, if altered, can lead to impacts on visual accommodation, leading to myopia or hyperopia, revealed that birds raised under blue light had a higher sphere index (0.625) than birds raised under white light (−0.020, *P* = 0.01). No differences were observed for cylinder (*P* = 0.62) and axis indices (*P* = 0.91, Table 4).

**TABLE 4:** Effect of different light color treatments on the refraction index of YPMX708 male broilers at 26 days of age (*n* = 18, Experiment 1, trial 2).

	Light treatment				
	Blue^1^	Green^2^	White^3^	*P*-value	SEM^4^
Sphere	0.625^a^	0.083^ab^	−0.020^b^	0.01	0.1032
Cylinder	0.604	0.458	0.667	0.62	0.0651
Axis	121.58	114.50	117.83	0.91	6.869

*^a, b^Means with common letters do not differ significantly (P ≤ 0.05).*

*^1^Blue light–dominant wavelengths 435–500 nm, peak at 455 nm.*

*^2^Green light–dominant wavelengths 500–565 nm, peak at 510 nm.*

*^3^White light–range of wavelengths from 380 to 780 nm.*

*^4^SEM = Standard error of the mean.*

### Spatial Vision

Light treatment had an impact on spatial vision. Birds raised under blue light took less time to approach the stimulus from a distance of 50 (*P* = 0.03) and 75 cm (*P* = 0.006) and had a higher success rate (choosing the right dispenser) at 100 cm (*P* = 0.03) than birds raised under white light (Table 5).

**TABLE 5:** The effect of light color treatments on spatial vision of YPMx708 male broiler chickens at 29 days of age (*n* = 24, Experiment 2).

Distance	50 cm			75 cm			100 cm			
	Light treatment									
	Blue^1^	White^2^	*P*-value	Blue^1^	White^2^	*P*-value	Blue^1^	White^2^	*P*-value	SEM^3^
Average time to approach (s)	8.6*^b^*	15.8*^a^*	0.03	5.9*^b^*	27.1*^a^*	0.006	11.7	13.2	0.42	2.27
Success rate (%)	91.7	91.7	1.00	91.7	66.7	0.11	91.7*^a^*	50*^b^*	0.03	4.69

*^a, b^Means with common letters do not differ significantly (P ≤ 0.05).*

*^1^Blue light–dominant wavelengths 435–500 nm, peak at 455 nm.*

*^2^White light–range of wavelengths from 380 to 780 nm.*

*^3^SEM = Standard error of the mean.*

## Discussion and Conclusion

As with light intensity and photoperiod, light wavelength can impact poultry behavior. As vision is important for birds, altering their ability to perceive their environment could potentially impact their welfare and affect behavior. Previous research has suggested that broilers raised under shorter wavelengths, including blue light, are less fearful and less stressed than broilers raised under green or white lights (see text foot note 1).

### Ocular Health

In response to a light stimulus, the normal pupillary light reflex results in the sphincter muscle of the iris contracting or relaxing, resulting in dilation or constriction of the pupil. Ideal vision is usually correlated to a good pupillary response to light (Bremner, 2004). A variation of the PLR test is the cPLR, where the device used for the test emits light of a specific wavelength. This may provide information about particular ocular changes and functions of photoreceptors (Rukmini et al., 2019). Given that each photoreceptor can be stimulated by a selective wavelength, the cPLR can assess the contribution of each photoreceptor to PLR (Rukmini et al., 2019). In the current study, the cPLR, performed on birds raised under white, blue or green lights, indicates that wavelength did not affect the function of photoreceptors. In an *in vitro* study conducted with mouse-derived cells, blue light led to more severe damage to photoreceptors than white and green light (Kuse et al., 2014). The differences found between this study and the current study may have been related to the particular characteristics of each species’ visual system or the light intensity used.

Anterior segment biomicroscopy was performed to screen for diseases in the anterior segment of the eye, including cataracts (Brown et al., 1987) and anterior uveitis (Rothova et al., 1987). Previous research has indicated that continual exposure to specific wavelengths, such as UV light regularly emitted by specific light sources such as fluorescent light bulbs, may cause cataracts (Walls et al., 2011). In humans, the development of cataracts can be caused by oxidative stress in corneal epithelial cells and may lead to apoptosis of the cornea (Ouyang et al., 2020). However, in our study, the anterior segment biomicroscopy assessment did not reveal any abnormalities in the birds tested, meaning that the use of wavelengths resulting in the blue, green or white lights emitted from LED bulbs had no impact on the anterior segment of the eye.

Indirect ophthalmoscopy was performed to assess the fundus of the eye, including the retina and pecten. Concerns in this area of the eye include damage to the photoreceptors present on the retina and impairment of retinal pigment epithelium (RPE) cells, as the normal function of both photoreceptors and RPE cells are necessary for the development of vision (Ouyang et al., 2020). The authors cited damage to these cells to be caused by blue light due to proliferation of the inflammatory response and DNA, lysosomes or mitochondria damage (Ouyang et al., 2020). Effects of shorter wavelengths, such as blue light, were cited in the literature studying mice (Nakamura et al., 2017) or cell cultures (Ozkaya et al., 2019). To our knowledge, no models using chickens were applied in research to investigate the effects of wavelength treatments resulting in blue light from LED bulbs on retinal damage. In the current study, light color did not have a clinically appreciable effect on the retina. As birds have a different spectral sensitivity than mammals, this could explain why no effects of short wavelength were found on retinal damage, even though the previously cited works reported microscopic retinal damage.

Normal intraocular pressure for broilers has been reported to be in the range of 16 mmHg (Prashar et al., 2007). In the current study, intraocular pressure remained within this range for all light treatments. In addition, no significant differences in IOP were found between birds raised under white, blue or green light. A previous study using cell cultures revealed blue light-induced necroptosis of the retinal ganglion cells’ mitochondria, which can lead to glaucoma (del Olmo-Aguado et al., 2016), even though our study focused on clinical exams vs. ultrastructure. However, as previously mentioned, chickens have a different spectral sensitivity, which could result in altered outcomes when exposed to shorter wavelengths.

### Eye Measurements

Normally, eye growth follows light-dark cues, with growth occurring during the photophase and decreased growth during the scotophase (Nickla, 2013; Leis et al., 2017). This can be correlated to hormones such as dopamine and melatonin (Nickla, 2013), which are released in a diurnal fashion (Nickla et al., 1998). Lack of these diurnal rhythms can directly lead to eye abnormalities, with the lack of control of eye growth resulting in adjustments to their shape or size. Turkeys and broilers exposed to longer daylength or low light intensity were found to have altered eye weights and dimensions, which could induce ocular diseases (Deep et al., 2013; Schwean-Lardner et al., 2013; Vermette et al., 2016). Buphthalmia, which is an enlargement of the eye globe, is usually related to elevated IOP and could lead to glaucoma or even blindness (Whitley et al., 1984). In some studies, eye shape appears to be altered by exposure to monochromatic light. For example, guinea pigs exposed to longer wavelengths had increased eye length (Liu et al., 2011). Depending on the species, eye growth is also related to exposure to different chromatic lights. In humans, eyes may display a longitudinal chromatic aberration, which causes wavelength defocus. In this scenario, eye growth is reduced when humans are under blue light compared to red light exposure (Rucker, 2019). Chickens, however, appear to be an exception for this pattern of response as eye growth did not differ when exposed to either UV, white or red light under low light intensities (Rohrer et al., 1992). In our study, wavelength treatments had no impact on eye shape and size when birds were exposed to blue, green or white light, suggesting that exposing broilers to these monochromatic colors had no or minor impact on physiology that could affect ocular growth.

### Refractive Index for Determination of Visual Accommodation

Results obtained from this test indicate the presence of emmetropia (normal vision), myopia, hyperopia or astigmatism. Light exposure is related to effects on emmetropization. In this dynamic process, the eye undergoes adjustments, so the image falls on the retina, instead of falling in front of the retina, leading to myopic defocus (nearsightedness) or behind the retina, leading to hyperopic defocus (farsightedness) (Nickla and Schroedl, 2012). Chicks raised under high light intensity develop hyperopia, whereas chicks raised under dim light acquire myopic refractive errors (Cohen et al., 2011). Previous work indicates that, in addition to illuminance, light wavelength appears to impact refraction. Wavelength defocus occurs due to a process called longitudinal chromatic aberration, which is a wavelength-depended refractive error, and the eye may compensate by altering growth, and therefore, exerting impacts on refraction (Liu et al., 2011; Smith et al., 2015; Rucker, 2019). As longer wavelengths (red light) are focused more posteriorly on the retina as compared to shorter wavelengths (blue light), it is expected that accommodation responds to chromaticity (Foulds et al., 2013). Therefore, exposing chicks to longer wavelengths results in myopic refraction (Foulds et al., 2013; Rucker, 2013), and in contrast, exposing chicks to shorter wavelengths results in hyperopic refraction (Foulds et al., 2013). The sphere values obtained in our study, which denote the eye’s refractive error, revealed that birds raised under blue light are slightly more hyperopic, or far-sighted, than birds raised under white light, which contains a range of wavelengths from short to long in its spectrum. The farther away from zero the sphere value is, the more hyperopic/myopic the refractive error. The results obtained were significantly different but numerically similar to an emmetropic eye; therefore, it is unclear if this level of refractive error would be large enough to result in significant visual impairment.

### Spatial Vision

To assess spatial vision, this study used visual acuity as an indicator *via* a “Grating Acuity Test”. This visual acuity test is commonly used for young children when language skills and letter identification are limited (Teller et al., 1986). Previous studies have successfully used grating stimulus to determine chicken visual acuity (DeMello et al., 1992).

To our knowledge, spatial vision has not been assessed in broilers with respect to varying wavelength treatments. In our study, birds reared under blue light approached the feeder sooner (50 or 75 cm) and were more successful at choosing the right feeder (100 cm), indicating that overall, they had better visual acuity than birds raised under white light. Chickens have a peculiar spectral sensitivity curve as compared to humans. These animals possess a significantly greater spectral sensitivity between 380 and 486 nm, corresponding to the violet and blue colors (Prescott and Wathes, 1999). This indicates that the common misconception in the poultry industry that birds would have impaired visual acuity under shorter wavelengths, such as blue, is incorrect. Such a misconception could be related to the comparison made using the pattern of human visual acuity and not considering the specificity of the avian species.

In conclusion, in our study, no impact of light color was observed on pupil light reflex, anterior segment biomicroscopy, indirect ophthalmoscopy, intraocular pressure, eye shape or size. The exposure to constant blue light resulted in minor differences in refraction. Birds raised under blue light were slightly more far-sighted than birds raised under white light. Birds raised under blue light approached a novel object with less delay when the object was near (50 and 75 cm) and were more successful when approaching a preferred object from a farther distance (100 cm), suggesting a superior visual acuity than birds raised under white light. The minor differences observed on refraction index and eye health may indicate that vision was similar for birds reared under blue and white light, except for spatial acuity. The improved spatial vision observed may partially explain the behavioral differences perceived when broilers are raised under blue light.

## Data Availability Statement

The raw data supporting the conclusions of this article will be made available by the authors, without undue reservation.

## Ethics Statement

The animal study was reviewed and approved by the Animal Care Committee of the University of Saskatchewan.

## Author Contributions

KS-L was the primary investigator. BR collected, analyzed and interpreted data, and drafted the manuscript. ML, MW, TS, TC, BF, NF, SG, and KS-L assisted with data collection and experimental design. All authors edited the manuscript.

## Conflict of Interest

BF, SG, and NF are employees by Aviagen Inc. The remaining authors declare that the research was conducted in the absence of any commercial or financial relationships that could be construed as a potential conflict of interest.

## Publisher’s Note

All claims expressed in this article are solely those of the authors and do not necessarily represent those of their affiliated organizations, or those of the publisher, the editors and the reviewers. Any product that may be evaluated in this article, or claim that may be made by its manufacturer, is not guaranteed or endorsed by the publisher.

## Footnotes

1 Remonato Franco, B., Shynkaruk, T., Crowe, T., Fancher, B., French, N., Gillingham, S., et al. (under review a). *Light Color and the Commercial Broiler: Effect on Behavior, Fear and Stress*.

2 Remonato Franco, B., Shynkaruk, T., Crowe, T., Fancher, B., French, N., Gillingham, S., et al. (under review b). *Does Light Color During Brooding and Rearing Impact Broiler Productivity?*

3 Remonato Franco, B., Shynkaruk, T., Crowe, T., Fancher, B., French, N., Gillingham, S., et al. (under review c). *Light Wavelength and its Impact on Diurnal Rhythms and Broiler Health*.
